# Omics Approaches for Understanding Biogenesis, Composition and Functions of Fungal Extracellular Vesicles

**DOI:** 10.3389/fgene.2021.648524

**Published:** 2021-05-03

**Authors:** Daniel Zamith-Miranda, Roberta Peres da Silva, Sneha P. Couvillion, Erin L. Bredeweg, Meagan C. Burnet, Carolina Coelho, Emma Camacho, Leonardo Nimrichter, Rosana Puccia, Igor C. Almeida, Arturo Casadevall, Marcio L. Rodrigues, Lysangela R. Alves, Joshua D. Nosanchuk, Ernesto S. Nakayasu

**Affiliations:** ^1^Department of Microbiology and Immunology, Albert Einstein College of Medicine, Bronx, NY, United States; ^2^Division of Infectious Diseases, Department of Medicine, Albert Einstein College of Medicine, Bronx, NY, United States; ^3^MRC Centre for Medical Mycology, University of Exeter, Exeter, United Kingdom; ^4^Biological Sciences Division, Pacific Northwest National Laboratory, Richland, WA, United States; ^5^Environmental and Molecular Sciences Laboratory, Pacific Northwest National Laboratory, Richland, WA, United States; ^6^Department of Molecular Microbiology and Immunology, Johns Hopkins Bloomberg School of Public Health, Baltimore, MD, United States; ^7^Laboratório de Glicobiologia de Eucariotos, Instituto de Microbiologia Paulo de Góes, Universidade Federal do Rio de Janeiro, Rio de Janeiro, Brazil; ^8^Departamento de Microbiologia, Imunologia e Parasitologia, Escola Paulista de Medicina-Universidade Federal de São Paulo, São Paulo, Brazil; ^9^Department of Biological Sciences, Border Biomedical Research Center, University of Texas at El Paso, El Paso, TX, United States; ^10^Laboratório de Regulação da Expressão Gênica, Instituto Carlos Chagas-FIOCRUZ PR, Curitiba, Brazil; ^11^Instituto de Microbiologia Paulo de Góes, Universidade Federal do Rio de Janeiro, Rio de Janeiro, Brazil

**Keywords:** extracellular vesicles, fungi, virulence, systems biology, proteomics, metabolomics, lipidomics, transcriptomics

## Abstract

Extracellular vesicles (EVs) are lipid bilayer structures released by organisms from all kingdoms of life. The diverse biogenesis pathways of EVs result in a wide variety of physical properties and functions across different organisms. Fungal EVs were first described in 2007 and different omics approaches have been fundamental to understand their composition, biogenesis, and function. In this review, we discuss the role of omics in elucidating fungal EVs biology. Transcriptomics, proteomics, metabolomics, and lipidomics have each enabled the molecular characterization of fungal EVs, providing evidence that these structures serve a wide array of functions, ranging from key carriers of cell wall biosynthetic machinery to virulence factors. Omics in combination with genetic approaches have been instrumental in determining both biogenesis and cargo loading into EVs. We also discuss how omics technologies are being employed to elucidate the role of EVs in antifungal resistance, disease biomarkers, and their potential use as vaccines. Finally, we review recent advances in analytical technology and multi-omic integration tools, which will help to address key knowledge gaps in EVs biology and translate basic research information into urgently needed clinical applications such as diagnostics, and immuno- and chemotherapies to fungal infections.

## Introduction

Cells secrete a variety of molecules to the extracellular milieu, from the smallest metabolites to large proteins and glycoconjugates. These secreted molecules range from toxic catabolites to cell communication molecules, virulence factors, and enzymes involved in nutrient acquisition. One particularly intriguing mechanism of secretion is the release of extracellular vesicles (EVs). Organisms from all kingdoms have been described to release EVs (Toyofuku et al., 2019; Woith et al., 2019; Rayamajhi and Aryal, 2020; Rizzo et al., 2020b). In fungi, EVs were first described and partially characterized in *Cryptococcus neoformans* (Rodrigues et al., 2007). Since the original description, different omics approaches have been instrumental for the characterization of EVs, from their putative mechanisms of biogenesis to their function in the fungal biology. In this article, we review the contribution of different omics approaches to study fungal EVs.

## Initial Characterization of Fungal Extracellular Vesicles

*Cryptococcus neoformans* EVs comprise a heterogeneous population of lipid bilayer vesicles, including pigment-containing, electron-lucid, electron-dense, and membrane-associated electron-dense vesicles. The first proteomic analysis of cryptococcal EVs led to the identification of 76 proteins involved in a variety of functions, including virulence, oxidative stress, unfolded-protein response, cellular metabolism, protein translation, signal transduction, cytoskeleton organization, and also proteins found in the plasma membrane (Rodrigues et al., 2008). Subsequent studies in *Histoplasma capsulatum* identified 206 EV-associated proteins with a similar diversity of functions to *C. neoformans* EVs, besides proteins involved in cell synthesis and remodeling (Albuquerque et al., 2008). Lipidomic analysis of *H. capsulatum* EVs led to the identification of 18 phospholipids, including phosphatidylethanolamine, phosphatidylcholine, and phosphatidylserine species (Albuquerque et al., 2008). These initial characterization of fungal EVs opened new questions about their biogenesis and roles in infection, along with their potential use for clinical and biotechnological applications, as discussed in the subsequent sections.

## Cellular Sites and Mechanisms of Extracellular Vesicle Formation in Fungi

**FIGURE 1:**
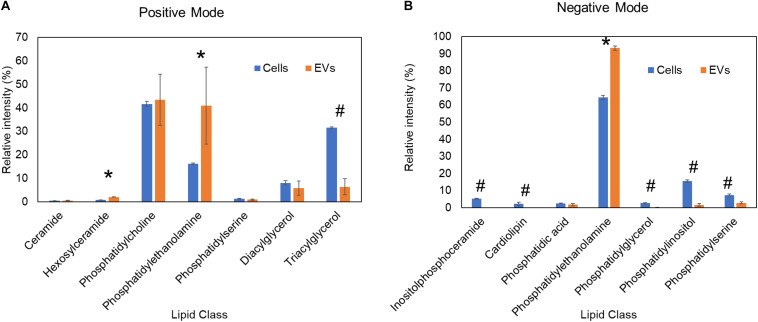
Comparative lipidomic analysis of *H. capsula*tum EVs and whole cells. Lipidomics data from previous publications (Burnet et al., 2020; Cleare et al., 2020) of yeast cells grown in F12 medium. Each lipid class was normalized by the total ion intensity of the identified lipid species. Lipid classes significantly (*p* ≤ 0.05 by Student’s *t*-test) enriched in extracellular vesicles and whole cells are shown by asterisks and hash signs, respectively. **(A)** Lipids quantified by mass spectrometry in the positive ionization mode. **(B)** Lipids quantified by mass spectrometry in the negative ionization mode.

The precise cellular sites and mechanisms of fungal EVs formation are still not fully defined. At first, there was some skepticism in the field that EVs may be products of dying cells whereby released lipids self-assembled into vesicles. Skepticism about EVs was also fueled by concerns of how such large structures could cross the cell wall, which was viewed as a rigid structure that would preclude vesicular transport. However, we have shown that heat-killed *C. neoformans* failed to secrete EVs (Rodrigues et al., 2007). With regard to the cell wall transit, recent studies have shown that this structure is easily penetrated by vesicles (Walker et al., 2018). To further investigate this issue, we compared lipidomic analysis data of *H. capsulatum* EVs (Cleare et al., 2020) and whole cells (Burnet et al., 2020). We found a significant depletion of the energy storage lipid, triacylglycerol, and of the mitochondrial lipid, cardiolipin, in EVs when compared with whole cells (Figure 1). The fact that EVs and whole cells have distinct lipid composition suggests they are formed by specific EVs biogenesis process(es), rather than being a product of cell death, and/or breakdown. Lipidomic analysis of EVs from two *Paracoccidioides brasiliensis* isolates of different phylogenetic groups showed differences in sterol and fatty acid composition of EVs when compared with whole cells, also suggesting the involvement of specific organelles in EVs biogenesis (Vallejo et al., 2012a). In addition, the deletion of the sterol biosynthesis gene Erg6 induced changes in the lipid and protein content of *C. neoformans* EVs, suggesting a role for sterols in EVs formation (Oliveira et al., 2020). Studies have shown that fungal EVs can originate from intracellular organelles, such as endosomes (Oliveira et al., 2010; Zarnowski et al., 2018; Zhao et al., 2019; Park et al., 2020), or at the plasma membrane (Rodrigues et al., 2000, 2013; Rizzo et al., 2020a). Morphological studies of *C. neoformans* showed structures resembling multivesicular bodies (MBVs) that can fuse to the plasma membrane, resulting in the release of intraluminal MBV vesicles into the fungal periplasm (Takeo et al., 1973). Those images suggested that populations of fungal EVs might correspond to exosomes, which are mammalian EVs released to the extracellular milieu by the fusion of MVBs to the plasma membrane (Raposo and Stoorvogel, 2013). This biogenesis pathway was supported by recent studies in *C. neoformans* (Park et al., 2020) and *Candida albicans* (Zarnowski et al., 2018), in which deletion of genes affecting MVB formation resulted in aberrant vesicles and/or decreased EVs production. In *Saccharomyces cerevisiae*, deletion of several regulators of either conventional or unconventional secretion resulted in alterations of EVs composition, as measured by proteomic analysis (Oliveira et al., 2010). In *C. neoformans*, the deletion of SEC6, a gene participating in the post-Golgi secretory pathway, also resulted in reduced EVs formation (Panepinto et al., 2009). In the filamentous fungus *Neurospora crassa*, GFP-localization of SEC-6, -5, -8, and -15 subunits of the exocyst complex each form a crescent just beyond the cluster of vesicles of the Spitzenkörper at an extending hyphal tip (Riquelme et al., 2014). The exocyst allows a physical linkage of the vesicle cluster to the apical membrane. The cellular events of these EVs is also a matter of debate, including fusion or vesicle budding and secretion (Rizzoli and Jahn, 2007; Miura and Ueda, 2018). These studies show that intracellular regulators of secretory pathways, such as the post-Golgi pathway, participate in fungal EVs formation, similar to what occurs in mammalian EVs.

As observed in protozoan parasites (Marcilla et al., 2014; Szempruch et al., 2016) and mammals (Raposo and Stoorvogel, 2013; Stahl and Raposo, 2019), fungal EVs can also be formed at the plasma membrane. Immunofluorescence of *C. neoformans* surface lipids revealed plasma membrane projections, suggesting that the plasma membrane could bud and release EVs (Rodrigues et al., 2000). The participation of the plasma membrane in fungal EVs formation was also shown using “wall-less” *Aspergillus fumigatus* cells (Rizzo et al., 2020a). Ultra-resolution microscopy analyses of these fungal protoplasts demonstrated the occurrence of EVs budding from the plasma membrane. Shedding of these plasma membrane-derived EVs was increased during cell wall synthesis, suggesting their participation in this process. Accordingly, protoplast EVs contain cell-wall polysaccharides and polysaccharide synthases, which are plasma membrane-associated enzymes (Rizzo et al., 2020a). These morphological and compositional studies support the presence of plasma membrane-derived EVs in fungi similar to the mammalian microvesicles (or ectosomes; Raposo and Stoorvogel, 2013; Stahl and Raposo, 2019). However, additional mechanisms of plasma membrane-derived EVs formation that differ from those described for mammalian microvesicles have been described in fungi. In *S. cerevisiae*, electron tomography studies revealed deep invaginations of the plasma membrane organized as two parallel membranes extending a few hundred nanometers toward the cell center. These structures can curve back to the cell surface, resulting in fusion with the plasma membrane and EVs formation (Rodrigues et al., 2013). Based on these observations, it has been proposed that fungal EVs might also originate from cytoplasmic content loading into a vesicle derived from the reshaping of the plasma membrane. The mechanisms behind this process are currently unknown, but they could represent a new pathway of EVs formation.

Overall, the studies described above show that fungi release exosome-like and microvesicle-like EVs, suggesting a conserved mechanism of EVs formation in lower and higher eukaryotes.

## Cell Wall Remodeling by Extracellular Vesicle Cargo

**FIGURE 2:**
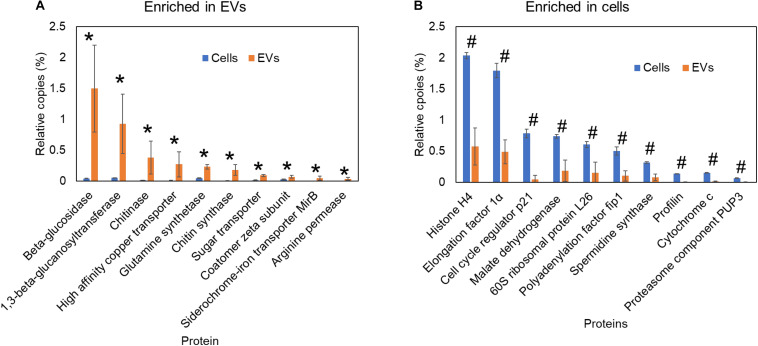
Comparative proteomic analysis of *Histoplasma capsulatum* extracellular vesicles and whole cells. Proteomics data from previous publications (Burnet et al., 2020; Cleare et al., 2020) of yeast cells grown in F12 medium. The relative copy number of proteins in cells or extracellular vesicles was calculated using intensity based absolute quantification (iBAQ) by normalizing to the total iBAQ of each sample. Proteins significantly (*p* ≤ 0.05 by Student’s *t*-test) enriched in extracellular vesicles **(A)** and whole cells **(B)** are indicated as asterisk and hash signs, respectively.

The cell wall is responsible for shaping fungal cells and for their resistance to diverse types of stress (Nimrichter et al., 2016). Nutrient availability, ambient pH, temperature, osmotic stressors, and other extracellular stimuli can lead to cell wall remodeling, which includes structural changes in their major components such as chitin, glucan, and glycoproteins (Nimrichter et al., 2016). The interplay between rigidity and plasticity of the cell wall is a key factor for fungal adaptation, survival, growth, and virulence (Nimrichter et al., 2016; Beauvais and Latgé, 2018). Although the cell wall synthesis and shaping are classically attributed to plasma membrane proteins, EVs might also play a role in this process (Vallejo et al., 2012b; Nimrichter et al., 2016; Ikeda et al., 2018; Zhao et al., 2019; Dawson et al., 2020). In *P. brasiliensis*, 60% of the non-covalently bound cell wall proteins, detected by proteomic analysis of two distinct isolates, have been described in fungal EVs (Longo et al., 2014). The EVs content can vary deeply depending on the growth conditions and fungal species (Vallejo et al., 2012a,b; Longo et al., 2014; Peres et al., 2015; Alves et al., 2019; Peres da Silva et al., 2019; Cleare et al., 2020). In addition, enrichment of cell wall remodeling enzymes is a conserved feature across fungal EVs (Vallejo et al., 2012b; Longo et al., 2014; Ikeda et al., 2018; Zhao et al., 2019; Dawson et al., 2020). Cell wall synthases or hydrolases have been found in EVs from *Candida auris, C. albicans*, *Cryptococcus deneoformans, Cryptococcus deuterogattii, C. neoformans*, *Fusarium oxysporum, P. brasiliensis*, *Sporothrix brasiliensis*, *S. cerevisiae*, *H. capsulatum*, and *Trichoderma reesei* (Vallejo et al., 2012b; Wolf et al., 2014; Ikeda et al., 2018; Bleackley et al., 2019; de Paula et al., 2019; Konečná et al., 2019; Zhao et al., 2019; Cleare et al., 2020; Dawson et al., 2020; Zamith-Miranda et al., 2020; Rizzo et al., 2020c). When we compared the proteomic data of *H. capsulatum* EVs (Cleare et al., 2020) with whole cells (Burnet et al., 2020), the results showed that EVs were highly enriched in cell wall synthases and hydrolases, such as β-glucanase, β-1,3-glucanosyltransferase, chitin synthase, and chitinase (Figure 2). In *S. cerevisiae*, supplementation with chitin synthase Chs3-enriched EVs rescued the growth of cells treated with the cell wall-targeting antifungal caspofungin, suggesting that the EVs cargo *per se* is sufficient to supply components for cell remodeling (Zhao et al., 2019). Enzymes that have already been described in fungal EVs are involved in evasion of the immune system by modifying cell wall epitopes. The presence of lactate or exposure to hypoxia induced β-1,3-glucan masking in *C. albicans*. This effect was mediated by the secreted exo-β-1,3-glucanase, Xog1, which has been described as an EVs cargo in proteomic studies (Nimrichter et al., 2016; Konečná et al., 2019; Childers et al., 2020; Dawson et al., 2020). However, the direct participation of EVs in this process still needs to be confirmed. The ability to actively secrete cell wall synthases and hydrolases through EVs in response to extracellular environmental signals could represent a new mechanism of cell wall remodeling, which could affect the exposure of epitopes during infection, consequently resulting in modulation of the immune response.

## Extracellular Vesicles and Antifungals

A seminal work by Andes laboratory showed that EVs were involved in biofilm formation in *C. albicans* (Zarnowski et al., 2018). Defective biofilm formation leads to an increased susceptibility to fluconazole. The authors performed gain-of-function experiments and showed that addition of wild-type EVs to biofilm-deficient strains restored biofilm formation and re-established fluconazole resistance. This work is akin to the previously mentioned work of EV-associated Chs3 restoring and rescuing cell wall defects and improving tolerance to antifungals (Zhao et al., 2019). Proteomic analyses performed in both studies showed that EVs carry a myriad of functional enzymes. Interestingly, it is possible that the delivery of a combination of enzymes in EVs allows for higher efficiency in cell wall remodeling.

## RNA Content in Fungal Extracellular Vesicles

Omic approaches caused a major impact in deciphering the RNA content carried by EVs, being most of the data available characterized using RNA-seq. In fungi, RNA export via EVs was originally described in *S. cerevisiae*, *C. albicans*, *C. neoformans*, and *P. brasiliensis* (Peres et al., 2015). Similar to what has been published for mammalian EVs, the fungal EVs transcripts were composed mainly of small RNA (sRNA) sequences of up to 250 nt. The most abundant classes were non-coding (nc)RNA sequences of the small nucleolar RNA (snoRNAs), small nuclear RNA (snRNAs), and tRNA types (Peres et al., 2015). Subsequent transcriptomics analysis of EVs from *H. capsulatum* (Alves et al., 2019) and *Paracoccidioides* (Peres da Silva et al., 2019) isolates revealed that the small ncRNAs were mostly represented by short 25-nt fragments that aligned to a specific region of a particular mRNA. The presence of anti-sense RNA fragments in EVs might have a role in gene silencing, maybe similarly to that of micro RNAs (miRNAs) and fungal exonic short interfering RNAs (Nicolás and Ruiz-Vázquez, 2013; Son et al., 2017). The fungus *Malassezia sympodialis*, a member of the human skin microbiota, also exports EVs containing 16 to 22 nucleotides long RNAs. However, *M. sympodialis* lacks an RNAi machinery, suggesting that this fungal species might bear an alternative miRNA production pathway (Rayner et al., 2017). In the dimorphic fungus *Pichia fermentans*, the length of EVs RNAs ranges from 25 to 130 nt. These transcripts are involved in the transition of this fungus from yeast to pseudohyphal morphology, which occurs in response to specific environmental conditions (Leone et al., 2018).

Although most of the fungal EVs transcripts were small ncRNAs, full-length mRNAs have also been found. In general, they corresponded to genes of metabolic pathways, transcription regulation, cell cycle, vesicle-mediated transport, cellular responses to stress, and translation, depending on the species and the species isolate studied (Peres et al., 2015; Alves et al., 2019; Peres da Silva et al., 2019). An *in vitro* translation experiment has shown that mRNAs carried by *P. brasiliensis* and *P. lutzii* EVs are functional (Peres da Silva et al., 2019). Based on these results, it is reasonable to speculate that EVs mRNAs can be transferred and translated into the host cell, possibly modulating gene expression that could benefit the pathogen infection and survival. In *Cryptococcus gattii*, the EVs derived from a virulent strain induced, inside macrophages, survival and proliferation of a less virulent strain, which would normally be cleared by the host cell. This phenotype was decreased by EVs pre-treatment with RNase, supporting a role for EV-associated RNAs in the transfer of virulence traits (Bielska et al., 2018). However, the nature of these RNAs and their mechanism of action still needs to be further investigated.

**FIGURE 3:**
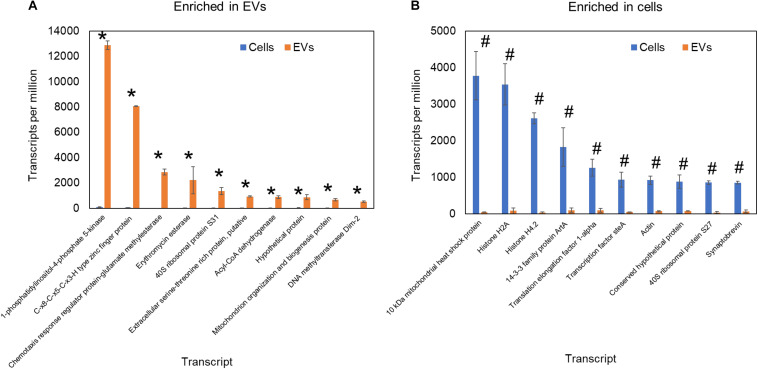
Comparative transcriptomic analysis of RNA content *H. capsulatum* extracellular vesicles and whole cells. The expression level is represented as transcripts per million. Transcripts significantly (*p* ≤ 0.05 by *t*-test) enriched in extracellular vesicles **(A)** and whole cells **(B)** are shown by asterisk and hash signs, respectively.

Regarding RNA loading into EVs, in mammals, the autophagy protein LC3 has been reported as a recruiter of RNA-binding proteins to these compartments (Leidal et al., 2020). In fungi, the composition of RNA in the EVs can be affected by alterations in the intracellular vesicle and secretion pathway. The knockout of Golgi reassembly and stacking protein in *C. neoformans* deeply affected the EVs RNA composition, suggesting a role of the Golgi in the EVs RNA loading (Peres et al., 2018). To further evaluate the existence of a specific mechanism of RNA loading into fungal EVs, we compared the published transcriptomics data of *H. capsulatum* EVs with that of the whole cell (Alves et al., 2019). We observed a striking enrichment of specific RNA sequences in EVs, while the most expressed RNAs in the cells were present only in trace amounts in EVs (Figure 3). These results support the hypothesis that RNA sorting to EVs is finely regulated. In addition, robust RNA-seq data comparing the transcriptomics of EVs with that of their corresponding *C. albicans* cells cultivated both under control and mild stress conditions. We observed that the EVs and the cell transcriptomics was distinct in all growth conditions and that the RNA content of both EVs and cells was modulated under the stress conditions analyzed (Leitão, 2017).

## Extracellular Vesicle Components as Disease Biomarkers and Cell Biology Markers

While the terms biomarkers and markers are often and inappropriately used interchangeably, they have distinct definitions. Biomarkers, by definition, are molecular signatures that can be used in the clinic to diagnose or predict the appearance or outcome of a disease (Strimbu and Tavel, 2010), whereas cell biology markers are molecules that can differentiate cell populations, cellular processes or cellular compartments (Zhang et al., 2019). The distinct composition of EVs from fungi and human cells make them good candidates for clinical diagnostic biomarkers and/or disease follow-up, i.e., for early assessment of chemotherapeutic outcomes and/or disease progression. Unfortunately, this subject has been understudied in fungi. In parasites, proteins from the murine malarial parasite *Plasmodium yoelii* have been detected in proteomics analysis of EVs from infected reticulocytes (Martin-Jaular et al., 2011). Similar findings have been recently observed in EVs isolated from plasma of a patient with chronic Chagas disease (Cortes-Serra et al., 2020). The use of EVs as biomarkers has been better explored in cancer biology. For instance, in prostate cancer, urinary EVs has been shown to carry RNAs that are signature of the disease outcome and are considered promising biomarker candidates (Pang et al., 2020). More recently, the proteomic analysis of EVs and particles from plasma and tissue showed that they can distinguish between normal and cancer cells with >90% sensitivity and specificity (Hoshino et al., 2020).

The discovery of specific markers of fungal EVs would have a major impact in cell biology research toward understanding their biogenesis, traffic, and function. In mammalian cells, good markers are the tetraspanins CD9, CD63, and CD81 (Andreu and Yáñez-Mó, 2014), which are currently unavailable for fungal EVs. In 2012, Vallejo et al. showed that 63% of the *P. brasiliensis* EVs proteins had orthologs described in EVs of *H. capsulatum, C. neoformans*, and *S. cerevisiae* (Vallejo et al., 2012b). Dawson et al. (2020) analyzed proteins that were enriched in EVs from three different strains of *C. albicans* when compared to the proteome of whole cells from the same strains. They found 47 commonly enriched proteins including Sur7, Evp1, and a variety of cell-wall synthesis and remodeling proteins. It should be noted, however, that the whole cell fraction that the authors analyzed did not include plasma membrane and, therefore, the results should be carefully considered (Dawson et al., 2020). Due to presence of cell-wall synthases and hydrolases in EVs from a variety of species (as discussed above) and since cell wall is conserved across the kingdom Fungi, it is reasonable to speculate that cell-wall synthesis and remodeling proteins could be a common marker for fungal EVs. The validation of these marker candidates and subsequent development of reagents may open new avenues to study the cell biology of fungal EVs, while biomarkers could be used for translational research as new diagnostic and prognostic tools.

## Extracellular Vesicles as Transporters of Virulence Factors

Early characterization of EVs from *C. neoformans* showed the presence of important previously described virulence factors, specifically, glucoronoxylomannan (GXM), melanin, monohexosylceramide, laccase, urease, and phosphatase (Rodrigues et al., 2008; Eisenman et al., 2009). So far, however, only a few studies have investigated the participation of fungal EVs during infection in vertebrates. Using a murine model of cryptococcosis, Huang and colleagues demonstrated that the co-injection of *C. neoformans* with EVs facilitated the yeast transversal of the blood-brain barrier and enhanced the disease development (Huang et al., 2012). In addition, EVs are associated with a higher fungal burden and an increased lesion diameter in early stages of sporotrichosis caused by *S. brasiliensis* (Ikeda et al., 2018). Over the last decade, a number of EVs proteomic analysis carried out in diverse pathogenic fungal species described the finding of proteins that are associated with fungal virulence, but the association between effect and EVs cargo is still speculative, due to the lack of appropriate molecular tools, especially genetically deficient strains for most fungal species and pharmacological inhibitors. These EV-associated proteins include hydrolytic enzymes involved in protein and lipid degradation, proteins that protect against host oxidative responses and other types of stress (Table 1).

**TABLE 1:** Effects and virulence factors in fungal EVs.

Fungus	*In vivo* effect of EVs	*In vitro* effect of EVs	Virulence factors carried by EVs	
*C. neoformans*	Pathogenesis	Stimulates cytokine production and antifungal activity in macrophages (Oliveira et al., 2010)	GXM	
	(Promotes brain infection)	Enhance adhesion and trans endothelial passage through endothelial cells activating lipid rafts (Huang et al., 2012)	Catalase and superoxide dismutase	
	Protection in *G. mellonella* and mice models of cryptococcosis (Rizzo et al., 2020c)	Melanin synthesis (Rodrigues et al., 2008)	Urease	
			Melanin/laccase	
*C. gattii*	nd*	Associated with virulence transference (Bielska et al., 2018)	Protein and RNA	
*C. albicans*	**Yeast EVs:**	**Yeast EVs:**	**Yeast EVs**	**Hyphae EVs**
	Protection in *G. mellonella* and mice models of candidiasis (Vargas et al., 2015, 2020)	Stimulates macrophages to produce NO and cytokines. Stimulates dendritic cells to produce cytokine and up-regulates MHCII and CD86 (Vargas et al., 2015)	**SAPs	***SAPs
			Als3 and 4	Als3
		Biofilm EVs:	PLB	PLB5 and PLC2
		Matrix production and biofilm drug resistance (Zarnowski et al., 2018)		****Ece1p
		Hyphae EVs:		
		Induced TNFα release in THP-1 cells (Martínez-López et al., 2020)		
*C. auris*	Induces adhesion to epithelium and activation of bone marrow-derived dendritic cells (Zamith-Miranda et al., 2020)	Adhesion to epithelial cells	Phosphatase	
			Peroxisomal catalase	
		Dendritic cell activation	Superoxide dismutase	
			SAP10	
			Phospholipases B and D	
			Thioredoxin Reductase	
*C. glabrata*	nd	nd	Phospholipase B	
*C. parapsilosis*	nd	nd	Lipase (Lip2)	
*C. tropicalis*	nd	nd	SAP	
			Hwp1-like protein	
			Lysophospholipase	
*H. capsulatum*	nd	Inhibits phagocytosis and killing by macrophages and impacts ROS generation (Matos Baltazar et al., 2016; Baltazar et al., 2018)	Catalase B, Superoxide Dismutase and a Thiol-specific antioxidant protein	
*P. brasiliensis*	nd	Induces production of proinflammatory mediators and the M1 polarization of macrophages.	gp43, 14-3-3, PbCdC42, catalase, superoxide dismutase	
		Enhance the fungicidal activity of macrophages (da Silva et al., 2016)		
*A. fumigatus*		Induces the production of TNF-alpha and CCL-2 by macrophages	Asp F3 and a putative thioredoxin reductase	
		Enhances the antifungal activity of macrophages and neutrophils (Souza et al., 2019)		
*A. flavus*	Protection in *G. mellonella* model of aspergillosis	Induces the production of inflammatory mediators (NO and cytokines) and the M1 polarization of macrophages. Enhance the fungicidal activity of macrophages (Brauer et al., 2020)	nd	
*S. brasiliensis*	Increase in fungal burden and lesion diameter in a mice model of sporotrichosis (Ikeda et al., 2018)	Enhancement of yeast phagocytosis and fungal burden in dendritic cells.	70 KDa-glycoprotein	
		Increase in cytokine production (IL-12p40 and TNF-alpha; Ikeda et al., 2018)		

*nd – not dertermined. **So far SAP 4 was the only member never reported in yeast *C. albicans* EVs. ***In hyphae EVs SAP2, 4, 5, 6, 7, 8, 9, and 10 were identified. ****Key proteins were found in hyphae EVs, but the toxin peptide candidalysin was not detected.

A combination of virulence factors is loaded in EVs from *Candida* species, including aspartyl proteases (SAPs), adhesion molecules, and lipases (Gil-Bona et al., 2015; Vargas et al., 2015; Karkowska-Kuleta et al., 2020; Martínez-López et al., 2020; Zamith-Miranda et al., 2020). In *P. brasiliensis*, six previously characterized virulence factors were detected in EVs (Vallejo et al., 2012b): gp43 (Torres et al., 2013), 14-3-3 (Marcos et al., 2016), catalase (Tamayo et al., 2017), cytochrome C peroxidase (Parente-Rocha et al., 2015), superoxide dismutase (Tamayo et al., 2016), and PbCDC42 (Almeida et al., 2009). In *C. neoformans*, proteomic studies revealed the presence of antioxidant enzymes such as catalase, superoxide dismutase, thioredoxin, thioredoxin reductase, and thiol-specific antioxidant protein (Rodrigues et al., 2008). Antioxidant proteins were found in *H. capsulatum* EVs, including catalase B, superoxide dismutase, and a thiol-specific antioxidant protein (Albuquerque et al., 2008), and *A. fumigatus*, of which Asp F3 and a putative thioredoxin reductase were found (Souza et al., 2019). Rodrigues and colleagues confirmed the urease and laccase activities in EVs released by *C. neoformans* (Rodrigues et al., 2008). Urease improves the survival of *C. neoformans* inside macrophages by modulating the phagosomal pH (Fu et al., 2018). EVs urease seems to be relevant during brain invasion (Huang et al., 2012). Laccase promotes pathogenesis of cryptococcal infections via multiple pathways: (1) synthesizing prostaglandins that may suppress local inflammatory responses (Erb-Downward et al., 2008); (2) inducing extrapulmonary dissemination to the brain (Noverr et al., 2004); (3) inhibiting the Th17-type cytokine response and neutrophils recruitment (Hansakon et al., 2020); (4) enhancing fungal survival in macrophages by mediating its escape through non-lytic exocytosis (De Oliveira Frazão et al., 2020); and (5) catalyzing the synthesis of melanin. However, the role in pathogenesis of these virulence factors present in EVs still need to be investigated.

Lipidomic, glycomic, and metabolomic studies of fungal EVs have also led to the identification of potential virulence factors. Lipidomic analysis comparing EVs from two *P. brasiliensis* isolates with different degrees of virulence showed distinct phospholipid and sterol contents, which might be associated with differential virulence. The more virulent Pb18 isolate had higher ergosterol to brassicasterol ratio than the Pb3 strain (Vallejo et al., 2012a), and considering the function of ergosterol in triggering macrophage pyroptosis it can represent a virulence mechanism (Koselny et al., 2018). EVs from *C. albicans* and *C. auris* carry a variety of lysophospholipids (Zamith-Miranda et al., 2020), which might be correlated with expression of phospholipases in these organisms. In fact, lysophosphatidylcholine well-characterized regulators of the host immune response (Soehnlein et al., 2009; Carneiro et al., 2013; Gazos-Lopes et al., 2014) and might have a role in candidiasis virulence. Monohexosylceramides have been found in EVs released by *H. capsulatum*, *P. brasiliensis*, *C. neoformans*, *C. albicans*, and *C. auris* (Rodrigues et al., 2007; Vallejo et al., 2012a; Cleare et al., 2020; Zamith-Miranda et al., 2020). Monohexosylceramide has been associated with *C. neoformans* ability to grow in neutral and basic pH (Rittershaus et al., 2006), and to promote *C. albicans* infection (Rittershaus et al., 2006). *N*-acetylsphingosine (also known as C2-ceramide), a regulator of the T-cell function (Menné et al., 2000; Detre et al., 2006), has been reported in EVs from *C. auris* (Zamith-Miranda et al., 2020) but its function in virulence still need to be investigated.

Peres da Silva et al. (2015) showed that *P. brasiliensis* and *P. lutzii* have a polysaccharide (or hydrolysis fragments) with glycogen structure and a galactofuranosylmannan oligomer as the main glycans in EVs. Small amounts of 1,3- and 1,6-cell wall glucans were also found. Indeed, β-1,3-glucan is an important cell wall inflammatory pathogen-associated molecular pattern. The study also included glycan and plant/mammalian lectin microarray profiling of EVs surface, revealing the presence of ligands of DC-SIGN receptors, exposed mannose and *N*-acetylglucosamine residues, and *N*-acetylglucosamine-binding lectin(s) that can potentially mediate interaction with the host. *P. brasiliensis* EVs carbohydrate content could indeed be implicated in the transcriptome modulation of murine monocyte-derived dendritic cells (Peres da Silva et al., 2015). A mechanism of fungal resistance to the host defenses by EVs has been shown in *C. neoformans* by shutting off the host inflammasome. Metabolomic analysis identified that the aromatic metabolite DL-Indole-3-lactic acid is secreted inside EVs, which in turn could impair the inflammasome activation by the host cells (Bürgel et al., 2020).

Overall, omics analyses have found a variety of molecules associated with virulence and regulation of the host immune response. However, how EVs promote virulence with their molecules still needs additional investigations.

## Host Response to Extracellular Vesicles

The presence of virulence factors and antigens suggests that fungal EVs could modulate the host response to infection. *A. flavus* and *P. brasiliensis* EVs enhance the phagocytosis of their respective yeast cells by macrophages (da Silva et al., 2016; Brauer et al., 2020). The EVs also induce the polarization of macrophages toward the proinflammatory M1 phenotype, which has been associated with high antifungal activity (da Silva et al., 2016; Brauer et al., 2020). Similar induction of pro-inflammatory cytokines has also been reported for EVs from *C. neoformans*, *C. albicans*, and *A. fumigatus* (Oliveira et al., 2010; Vargas et al., 2015; Souza et al., 2019). Conversely, EVs can also impair specific host responses. EVs released by *M. sympodialis* drive the production of the cytokine IL-4 by human peripheral blood mononuclear cells (Gehrmann et al., 2011). It is believed *M. sympodialis* EVs have a function in allergic responses. Proteomic analysis identified that 10 of 13 previously characterized allergens produced by the fungus are present in EVs. Two of these proteins were enriched in EVs as compared to fungal cells (Johansson et al., 2018).

Host immune factors, such as antibodies, can induce changes in the composition of EVs (Matos Baltazar et al., 2016; Baltazar et al., 2018). Incubation of *H. capsulatum* yeasts with antibodies against HSP60 (heat-shock protein 60), a protein enriched in cell wall and EVs, significantly changed the EVs cargo (Matos Baltazar et al., 2016; Baltazar et al., 2018). This treatment led to an increase in protein content and the virulence factor urease, suggesting a counteraction of the fungal resistance mechanisms against the host defenses (Matos Baltazar et al., 2016). Moreover, the EVs from antibody-treated *H. capsulatum* have an inhibitory effect on phagocytosis by macrophages (Baltazar et al., 2018). EVs from *S. brasiliensis* enhanced phagocytosis of the respective cells and increased the fungal burden in dendritic cells (Ikeda et al., 2018).

As potential targets for immunotherapies, EVs from *C. neoformans, C. albicans and A. flavus* have been shown to elicit at least a partial protection in the moth *Galleria mellonella* or in mice (Vargas et al., 2015; Colombo et al., 2019; Brauer et al., 2020; Rizzo et al., 2020c). The EVs cargo seems to be crucial to modulate this response. The presence of GXM and sterylglucosides in EVs reduces the protective effects of EVs in *G. mellonella* (Colombo et al., 2019). In macrophages, *C. neoformans* EVs containing GXM trigger a lower antifungal immunological response compared to EVs from a strain with reduced GXM production (Oliveira et al., 2010). *C. albicans* EVs activate murine macrophages and dendritic cells, inducing a protective immune response in immunosuppressed mice (Vargas et al., 2015; Vargas et al., 2020). Proteomic analysis revealed several candidates that could be involved with this response, including immunogenic proteins MP65 and Bgl2 (Nisini et al., 2001; Gil-Bona et al., 2015). Purified MP65 induces the expression of the antigen presentation protein MHC-II and co-stimulatory molecules, such as CD86, in dendritic cells (Pietrella et al., 2006). Similarly, the endo-β-1,3-glucanase Bgl2 has been tested as a vaccine candidate with promising results (Gil-Bona et al., 2015). These studies highlight the potential of fungal EVs as candidates for vaccine development.

## Extracellular Vesicles and Biomass Degradation

Biomass degradation by fungi is of major importance for agriculture and for biotechnological purposes. In agriculture, fungal infections and degradation of plants can cause major losses, while in biotechnology fungi can be used to convert biomass into biofuels or other bioproducts of economic value (Almeida et al., 2019; Oh and Jin, 2020). *F. oxysporum* is an environmental fungus that attacks cotton crops leading to significant losses in productivity (Gordon, 2017). *F. oxysporum* EVs induce damage on leaves from cotton and *Nicotiana benthamiana*, a close relative of tobacco, but its spores or hyphae do not cause the same damage. This effect, however, is not intrinsic to fungal EVs, since EVs from *S. cerevisiae* are not phytotoxic (Bleackley et al., 2019). Similarly, the wheat pathogen *Zymoseptoria tritici* (Hill and Solomon, 2020) produces EVs, which may be a part of its transition from apoplastic, non-symptomatic growth to necrotrophy of wheat. While some carbon-active enzymes associated with EVs were produced under media-grown conditions, additional *Zymoseptoria* effectors are expected *in planta*.

In biotechnology, degradation of cellulose from plants releases carbon for biofuel production (Oh and Jin, 2020). Soluble sugars released by fungal enzymes are built into ethanol, lipids, secondary metabolites, or other bioproducts accessible by fungal fermentation. *T. reesei* is a fungus that produces large amounts of cellulolytic enzymes. The proteomic analysis of EVs produced by this fungus revealed that enzymes with cellulase activity are exported through EVs. The cellulolytic activity of EVs from *T. reesei* is induced when the fungus is cultivated in the presence of cellulose, suggesting that cargo contained in fungal EVs is altered according to the environment (de Paula et al., 2019). Engineering yeasts for direct cellulosic degradation into ethanol producing yeasts could lead to important gains in biofuel production (Oh and Jin, 2020). In further support of environmental cues regulating EVs, growth conditions of submerged media vs. solid state fermentation (SSF) have shown that *Aspergilli* produce different secreted protein profiles. *A. oryzae* (Oda et al., 2006) secreted 4-6x more protein in SSF. *A. brasiliensis* ATCC9642 in SSF produces several differentially expressed proteins which lack secretion signals, suggesting an alternative route of secretion such as EVs (Volke-Sepulveda et al., 2016). Dissecting the molecular trafficking of EVs formation would create a novel compartment for enzyme delivery, or bioproduct collection from a culture without specialized transport proteins.

## New Methodologies and Instrumentation

The small size and scarce amount of material obtainable in preparations of EVs represent a major analytical challenge. However, advances in omics technologies have immensely improved the sensitivity, throughput, and robustness of the measurements, leading to a more comprehensive characterization of the EVs molecular composition. For instance, back in 2008, EVs proteomic analysis in *C. neoformans* led to identification of 76 proteins (Rodrigues et al., 2008). Current high-resolution tandem mass spectrometry (HR-MS/MS)-based approaches (Lesur and Domon, 2015), including nanoflow liquid chromatography coupled to HR-MS/MS (nanoLC-HR-MS/MS; Sanders and Edwards, 2020), allow to identify and quantify over 2,000 proteins in fungal EVs (Zhao et al., 2019; Cleare et al., 2020). In this section, we will cover recent technological advances and their current impact and perspectives in analyzing fungal EVs.

### RNA-seq

The RNA yield recovered from EVs is quite variable when we compare samples from different origins and that have been isolated using distinct protocols. For fungal cells, there are many media and growth conditions that can affect the number of EVs obtained and, consequently, the amount of RNA. Usually, the EVs RNA yield for *C. neoformans*, *C. albicans*, *P. brasiliensis*, and *H. capsulatum* ranges from 1 to 15 ng when the EVs are isolated from culture supernatant after two ultracentrifugation steps, corresponding to EVs enrichment and washing (Peres et al., 2015). Growing fungi in solid media, instead liquid cultures, can improve the EV RNA recovery yield to up to 50 ng, as shown for *C. neoformans* and *C. gattii* preparations (Reis et al., 2019).

In the recent years, the next-generation sequencing has emerged as a robust tool to resolve the diversity of RNA sequences in EVs. RNA-seq has enabled major advances in the analysis of EVs RNAs, allowing the identification of low input amounts of distinct RNA populations (Kim et al., 2017). Such technology allows the comparison of EVs RNA across samples generated under a variety of experimental designs, such as different growth conditions, stresses, or even interspecies studies (Mateescu et al., 2017; Yeri et al., 2018). Given that small RNAs are highly enriched in EVs, most of the library construction protocols focus on the fractioning of this RNA population. There are many kits available, but most of them follow similar procedures, involving multiple steps for the small RNA purification (Giraldez et al., 2018). Overall, we believe that RNA-seq will have a major impact in identifying EVs RNAs that could have a role in the host-pathogen interaction. Especially those sequences regulating expression of mRNAs in recipient cells and consequently, affecting the host immune response or the fungal pathogenicity.

### Recent Advances in Sample Preparation for Mass-Spectrometry-Based (multi)Omic Analysis

The eternal challenge of an analytical chemist is to improve sensitivity, precision, and speed of the instrumentation, enabling the accurate analysis of trace amounts of samples in large scale. In this context, the recent developments in single-cell analysis might have a major impact in analysis of EVs as most of the procedures are easily adaptable for analyzing EVs. An important concept is to keep the volumes and contact surfaces as small as possible during the sample preparation and analysis to reduce losses due to contact absorption. Online sample preparations can eliminate losses associated with pipetting and sample transfer. One of such techniques, called SNaPP (simplified nanoproteomics platform for reproducible global proteomics), allows the preparation and analysis of proteomic samples from nanograms of proteins (Huang et al., 2016). SNaPP has been successfully used to analyze Nipah virus-like particles (Johnston et al., 2019), which are secreted structures that share many characteristics with EVs, including size and the presence of a lipid membrane. Therefore, SNaPP has a great potential to be used for preparing EVs samples. Another technique to prepare small scale samples, but that has not yet been applied to study EVs, is the nanoPOTS (nanodroplet processing in one pot for trace samples; Zhu et al., 2018). nanoPOTS uses a microfluidic robot to prepare samples in nanoliter volumes, virtually eliminating any losses associated with absorption of proteins and peptides to the walls of pipettes and tubes. This technique has enabled to identify and quantify up to 2,500 proteins in single-cell proteomics analysis (Tsai et al., 2020), therefore, it might have an impact on analyzing EVs in the future.

Another analytical challenge in EVs studies is to obtain multiple omics measurements from the same samples. Having multiple measurements from the same samples is highly desirable since it decreases variability between datasets and efforts/costs associated with EVs preparation. Solvent phase separation-based extraction of metabolites, lipids, and proteins have been developed (Coman et al., 2016; Nakayasu et al., 2016). While the simultaneous metabolite, protein, lipid extraction (SIMPLEX) approach is based on methyl tert-butyl ether, methanol, and water; the metabolite, protein, and lipid extraction (MPLEx) technique uses a mixture of chloroform, methanol, and water for phase separation. To our knowledge, there are no papers in the literature reporting the analysis of EVs using SIMPLEX to extract the samples, even though its potential has been highlighted in a few review articles (Rosa-Fernandes et al., 2017; Ramirez et al., 2018). MPLEx, on the other hand, has been successfully used to analyze EVs from *H. capsulatum*, *C. auris*, and from the Gram-positive bacterium *Listeria monocytogenes* (Coelho et al., 2019; Cleare et al., 2020; Zamith-Miranda et al., 2020).

### Small Scale and Increased Throughput of Lipidomics, Metabolomics and Proteomics

When compared to conventional LC-MS/MS, advanced multidimensional analytical platforms such as LC-ion mobility spectrometry (IMS)-MS/MS, which combines liquid chromatography, ion mobility spectrometry, and tandem mass spectrometry, offers improvements in separation peak capacity, dynamic range, number of analytes detected, and quality of mass spectra (Baker et al., 2010; Rainville et al., 2017). Thus, the addition of IMS allows for improved coverage of metabolites, lipids, and proteins in EVs. Ion mobility also allows to separate isobaric molecules and enables detailed characterization of lipid molecular structure, including double-bond location, *cis*/*trans* orientation, and *sn*-positions of alkyl and/or acyl chains, none of which are possible using conventional LC-MS technologies (Zheng et al., 2018). Poad et al. (2018) have previously demonstrated the successful incorporation of the online ozonolysis into the existing LC-IMS-MS/MS instrumentation to enable characterization of double bonds in lipid standards. The IMS dimension significantly improves assignment of the ozonolysis products to their precursor ions.

One important challenge of the metabolomics and lipidomics fields is the reliable identification of molecules. Most of the identifications to date are validated by comparing MS/MS spectra and chromatographic elution profiles to *bona fide* standards. The inclusion of IMS separations opens a new perspective in the identification of small molecules without standards. The separation in IMS can be predicted with high precision (<5% error; Colby et al., 2019). Therefore, using multiple pieces of information, i.e., high mass accuracy, ion mobility separation, and tandem mass fragmentation might allow the identification of molecules without the need to validate them against standards, which would make metabolomic and lipidomic analyses much faster. A major bottleneck for metabolite identification is the reliance on reference databases that are constructed from authentic reference materials. This approach is highly limiting due to the cost and availability of authentic standards. Both commercial and publicly available reference databases currently only represent a small fraction of the possible metabolites that exist in biological systems (Dobson, 2004). The recent emergence of standards-free methods based on *in silico* methods such as quantum chemistry, machine learning, and deep learning have enabled accelerated building of very large reference libraries (Colby et al., 2020) with better coverage of the known chemical space, which was not feasible using authentic standards. Standards-free metabolomic approaches can therefore provide comprehensive coverage of the metabolome in EVs analyses, accelerating the discovery of small molecules and improving our understanding of their biological functions.

The low throughput of EVs analysis is a major challenge to conduct clinical studies to assess the EVs potential as a disease biomarker or to better understand their function in human health. One way to increase the throughput of analysis is by multiplexing samples. For proteomic analysis, isotope labeling or isobaric labeling can be used to multiplex samples. In this context, isobaric tags, such as isobaric tags for relative and absolute quantitation (iTRAQ) and tandem mass tag (TMT), allow multiplexing up to 16 samples (Li et al., 2020), thus increasing the speed of sample analysis. Another advantage of multiplexing is the relative increase in sample amount, as compared to analyzing them individually, providing gains in sensitivity. However, such techniques are not available for lipidomic and metabolomic analyses. Therefore, the gain in throughput of such analyses relies on reducing the time needed for the analysis of each sample. Ion mobility spectrometry is not only fast (milliseconds per scan), but also adds another dimension of separation to the LC-MS analysis, which allows to shorten the chromatographic separation time without compromising the depth of coverage. One of such concepts has been developed by the Evosep company (Odense, Denmark). The Evosep chromatographic system performs offline sample solid-phase extraction (SPE) and elution gradient, which is accumulated in a sample loop. During the analysis, the flow passes through the loop carrying the samples to an analytical column and subsequently to the mass spectrometer. This process eliminates the time needed for column regeneration and re-equilibration, consequently increasing the number of samples that can be analyzed in a day. The Evosep system coupled to IMS-MS allows to analyze up to 60 samples a day with a coverage that exceeds 5,000 proteins or more than 1,000 proteins with 5-min separation gradients (Meier et al., 2018; Bekker-Jensen et al., 2020). For the analysis of lipids and metabolites, due to the overall lower intrinsic complexity of their structures and MS fragmentation profile, as compared to peptides, it is possible to even reduce the time needed for the analysis each sample by an IMS-MS-based approach, as recently proposed by Zhang et al. (2016). These authors used an automated SPE platform coupled to IMS-MS to analyze metabolites and xenobiotics in the human urine (Zhang et al., 2016). They employed six SPE columns with a broad range of chemical properties that allowed them to capture distinct sets of molecules. In this configuration, each sample is run 6 times, or 12 times if analyzed in both positive and negative modes, but each cycle only takes 10 s. Therefore, each sample only takes about 2 min to be analyzed, allowing the analysis of hundreds of samples a day.

### Integration of Multi-Omics Data

The combination of multiple omics measurements allows to obtain a much deeper view of the EVs composition. Despite the challenges associated with processing large amounts of data, there are excellent tools to handle such tasks, which we will not cover in this review. Integrating the results of each omics measurement provides an opportunity to understand much deeper details and the processes occurring in EVs and cells. Despite some interpretations might be limited in EVs, the integration of proteomics with metabolomics and lipidomics, for instance, might show consistent changes in the levels of enzymes, substrates, and products. We have applied this approach to study whole cells of the multidrug-resistant fungus *C. auris*, which showed consistent changes in the levels of enzymes with their respective lipid and metabolite products in drug-resistant strains (Zamith-Miranda et al., 2019). Further integration of such data with RNA-seq results may further provide insights into the transcriptional and post-transcriptional regulation of genes. Nematode parasites, for instance, secrete miRNAs via EVs that target and modulate the expression of host immune factors (Buck et al., 2014).

## Concluding Remarks, Major Knowledge Gaps and Perspectives

Omic approaches have been contributing to the field of fungal EVs since the initial characterization of these extracellular “organelles.” Proteomic, lipidomic, metabolomic and RNA-seq technologies enabled a detailed characterization of the molecular composition of the fungal EVs, bringing new insights into their biogenesis, and biological and pathophysiological functions. Multiple omics analyses of mutant strains defective in different secretory pathway components have shown the participation of the Golgi complex in EVs biogenesis. In terms of EVs functions, omics analyses have played a key role in showing the participation of EVs in cell-wall remodeling and downstream function in antifungal resistance. Fungal EVs have also been shown to carry a variety of virulence factors, which opens new perspectives on how they are delivered to and interact with the host. Here are some major knowledge gaps in the field and how omics can contribute to close such gaps:

• *Biogenesis and EVs populations*. Despite numerous efforts of the field, the question regarding different EVs populations, their composition, and biogenesis processes is still open. Novel EVs purification techniques, such as differential centrifugation and affinity purification, might allow to separate different EVs populations, which in combination with genetics can lead to the identification of biogenesis pathways. Omics analyses can contribute by comprehensively characterizing the composition of different EVs populations.
• *Markers and biomarkers*. The absence of markers and biomarkers is a major impairment to perform cell biology and clinical studies on EVs, respectively. Ideally, would be to perform omics analysis of dozens of fungal species to identify EVs markers, whereas for developing clinically relevant biomarkers it often requires the analysis of hundreds to thousands of samples from multiple cohorts. Therefore, faster and more sensitive techniques will empower such studies.
• *Mechanisms of virulence*. Omics analysis will continue to detect or identify new virulence factors and their mechanisms. A major bottleneck is to study their mechanisms of action in host cells and animal models. We believe that techniques, such as co-affinity purification, followed by nanoLC-HR-MS/MS or an orthogonal analytical approach such as IMS-MS/MS, may have a pivotal role in identifying targets of virulence factors in the host cells, leading to a better understanding of the pathogenic mechanisms.
• *Structure-function relationship of fungal EVs molecules*. Structure-function relationship is another major gap in fungal EVs research. Thus far, most aforementioned studies that have identified fungal EVs molecules by proteomic, lipidomic, transcriptomic, and other omic approaches are descriptive in nature. In the coming years, investigators in the field should make greater strides to study the structure-function relationship of some of these fungal molecules, particularly those that have known or potential bioactivity, based on published data on fungi or other pathogen(s). This would require considerable improvement in (a) gene expression and knockout techniques for fungi; (b) purification and structural analysis of fungal molecules; and (c) chemical and/or enzymatic synthesis of fungus-specific molecular targets such as lipids, glycoconjugates, and metabolites.

Technological advances and Science are highly dependent on each other to progress, which is not different for EVs biology and omic analyses. We foresee that advances in omic technologies will continue having major impact in studying EVs biology.

## Author Contributions

All authors contributed to the literature review, writing of the manuscript and revision, and editing of the manuscript. All authors revised and approved the final version of the manuscript.

## Conflict of Interest

The authors declare that the research was conducted in the absence of any commercial or financial relationships that could be construed as a potential conflict of interest.
